# The effect of stroke on foot biomechanics; underlying mechanisms and the functional consequences

**DOI:** 10.1186/1757-1146-7-S1-A18

**Published:** 2014-04-08

**Authors:** Saeed Forghany, Christopher J Nester, Sarah F Tyson, Stephen Preece, Richard K Jones

**Affiliations:** 1 Centre for Health Sciences Research, University of Salford, UK; 2 Musculoskeletal Research Centre, School of Rehabilitation Sciences, Isfahan University of Medical Sciences, Iran; 3 Stroke Research Centre, School of Nursing Midwifery and Social Work, University of Manchester, UK

## Background

Although approximately one-third of stroke survivors suffer abnormal foot posture and this can influence mobility [1], there is very little objective information regarding the foot and ankle after stroke.

## Objective

The aim of this study was to investigate foot and ankle biomechanics, multi-segment foot kinematics and plantar pressure distribution in people with stroke and explore the possible causes and consequences of any abnormalities.

## Methods

In a single assessment session, mobility limitations (Walking Handicap Scale), multi-segment foot and ankle kinematics and plantar pressure distribution, electromyography of major posterior and anterior leg muscles, plantarflexor stiffness, plantarflexor and dorsiflexor strength and spasticity, and ankle proprioception were measured during stance phase of walking in 20 mobile chronic stroke survivors and 15 sex and age-matched healthy volunteers. Independent t-tests were used to compare the data for the stroke and healthy control groups. Multiple linear and binary logistic regressions were used to determine possible causes and functional consequences, respectively.

## Results

Compared to the healthy volunteers, the stroke survivors demonstrated consistently reduced range of motion across most segments and planes, increased pronation and reduced supination, disruption of the rocker and the timing of joint motion (Table 1). A more pronated foot prior to heel off and a less supinated foot during propulsion were biomechanical abnormalities significantly associated with limited functional ability. Soleus spasticity, excessive coactivity of tibialis anterior and medial gastrocnemius, and soleus, and plantarflexor stiffness were associated with these biomechanical abnormalities.

**Table 1:** Mean and standard deviation movements of each foot segment in each plane

Parameter	Stroke survivors	healthy volunteers	P value (95%CI)
**REARFOOT MOTION - SAGITTAL PLANE**			

**Range of movement during initial plantarflexion**	3.3° ± 2.1°	5.4° ± 2.5°	P < 0.007 (-3.6 to -0.6)
**Range of plantarflexion during late stance**	11° ± 4.6°	15.6° ± 4.5°	P < 0.003 (-7.5 to -1.7)

**REARFOOT MOTION - FRONTAL PLANE**			

**Total range of movement**	8.9° ± 3.2°	12° ± 3.3°	P < 0.006 (-5.1 to -0.9)
**maximum eversion**	3.5° ± 2.1°	2.3° ± 1.5°	P < 0.05 (-0.06 to 2.3)
**Range of inversion during late stance**	8.8° ± 3.4°	12° ± 3.4°	P < 0.006 (-5.3 to -1.0)

**REARFOOT MOTION – TRANSVERSE PLANE**			

**Total range of movement**	6.4° ± 2.6°	9.0° ± 4.9°	P < 0.04 (-5.1 to -0.09)
**Maximum abduction**	1.3 ± 2.7°	3.3° ± 3.2°	P < 0.05 (-3.8 to -0.03)
**Range of movement during the adduction phase**	6.1 ± 2.9°	9.0° ± 4.9°	P < 0.03 (-5.5 to -0.3)

**FOREFOOT MOTION - SAGITTAL PLANE**			

**Range of final plantarflexion phase**	1.9° ± 2.1°	4.6° ± 3.3°	P < 0.008 (-4.8 to -0.8)

**FOREFOOT MOTION – TRANSVERSE PLANE**			

**Range of the final adduction phase**	1.3° ± 1.8°	3.1° ± 1.9°	P < 0.009 (-3.1 to -0.5)

## Conclusions

Our findings highlight structural and movement deficiencies in foot joints in all three planes which does not support common clinical practices that focus on sagittal ankle deformity and assumed excessive foot supination. Some of foot abnormalities were associated with limitation in functional ability. Spasticity, the hyperexcitability of the stretch reflex, was a common predictor of all dynamic biomechanical abnormalities limiting functional ability. Biomechanical abnormalities and neuromuscular impairments of foot and ankle can be modified using physical therapies and future interventions might better target specific aspects of foot function and thereafter improve functional ability post stroke.
